# Supplementary material for: In silico analysis of local RNA secondary structure in influenza virus A, B and C finds evidence of widespread ordered stability but little evidence of significant covariation
Source: Sci Rep. 2022 Jan 10;12:310. doi: 10.1038/s41598-021-03767-x (PMC8748542; doi:10.1038/s41598-021-03767-x)

IAV_seg5_1_1

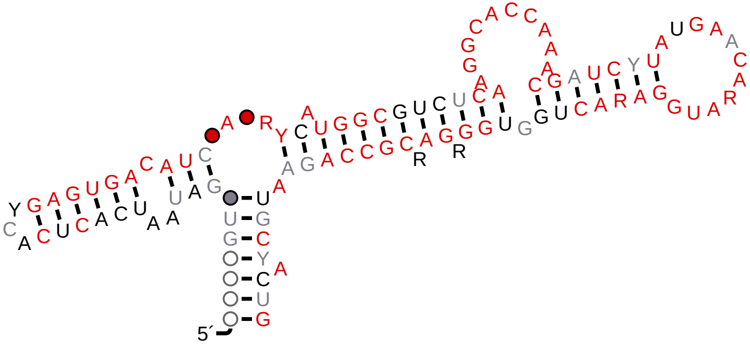

Supplement: Supplementary file 1 — Supplementary Information. [file 41598_2021_3767_MOESM1_ESM.zip › Supplemental Files/Supplemental File 3.zip!/Supplemental File 3/CM-AAll/pdf/IAV_seg5_1_1.R2R.sto.pdf]
